# Investigating trade-offs between ovary activation and immune protein expression in bumble bee (*Bombus impatiens*) workers and queens

**DOI:** 10.1098/rspb.2023.2463

**Published:** 2024-01-24

**Authors:** Alison McAfee, Abigail Chapman, Grace Bao, David R. Tarpy, Leonard J. Foster

**Affiliations:** ^1^ Department of Biochemistry and Molecular Biology, Michael Smith Laboratories, University of British Columbia, Vancouver, British Columbia, Canada V6T1Z4; ^2^ Department of Applied Ecology, North Carolina State University, Raleigh, NC 27695-7617, USA

**Keywords:** resource allocation, trade-off, immunity, reproduction, bumble bees, proteomics

## Abstract

Evidence for a trade-off between reproduction and immunity has manifested in many animal species, including social insects. However, investigations in social insect queens present a conundrum: new gynes of many social hymenopterans, such as bumble bees and ants, must first mate, then transition from being solitary to social as they establish their nests, thus experiencing confounding shifts in environmental conditions. Worker bumble bees offer an opportunity to investigate patterns of immune protein expression associated with ovary activation while minimizing extraneous environmental factors and genetic differences. Here, we use proteomics to interrogate the patterns of immune protein expression of female bumble bees (*Bombus impatiens*) by (i) sampling queens at different stages of their life cycle, then (ii) by sampling workers with different degrees of ovary activation. Patterns of immune protein expression in the haemolymph of queens are consistent with a reproduction–immunity trade-off, but equivalent samples from workers are not. This brings into question whether queen bumble bees really experience a reproduction–immunity trade-off, or if patterns of immune protein expression may actually be due to the selective pressure of the different environmental conditions they are exposed to during their life cycle.

## 1. Introduction

The ability to reproduce and withstand disease exposure is central to every species' survival. As both tasks are energetically demanding processes, the two capabilities are expected to be at odds [1], and much data support the existence of a reproduction–immunity trade-off in insects (reviewed in [2]). This trade-off is especially apparent among females, which invest significantly more resources into offspring production than males (although males can also be subject to such a trade-off [3]). In females, there are numerous examples of negative relationships between mating and immune function [4–8], and between immune stimulation and fecundity [5,9–17]. However, there are also some examples where the converse is true; highly fecund individuals may also have superior immune systems [18–20], and mating can cause immune activation [21–24]. This discrepancy suggests that reproduction and immune function do not always exist in a zero-sum relationship, and other factors may be involved in shaping these outcomes.

Eusocial insects appear to defy the typical trade-off between lifespan and fecundity—highly fecund females are also the longest-lived individuals [25–27]—and there is evidence for [4,28,29] and against [19,30,31] a reproductive–immunity trade-off, depending on the system. This makes hymenopterans an intriguing group of insects to interrogate, since the factors shaping their life histories are often complicated by their sociality and are not always subject to the same biological constraints as solitary insects, from which the majority of evidence for a reproductive–immunity trade-off has been derived [2]. For example, in honey bees (*Apis mellifera*) and black garden ants (*Lasius niger*), the long lifespan of queens is linked to high abundance of vitellogenin, but the hormonal pathways regulating vitellogenin production are sometimes inconsistent with those in solitary insects [31,32]. In honey bee queens, for instance, treatment with a juvenile hormone analogue (Methoprene) decreases vitellogenin expression [32], but in *Drosophila*, juvenile hormone treatment increases vitellogenin expression [33]. Some authors suggest that conserved hormone signalling pathways are ‘rewired' in honey bees to produce the large differences in lifespan and reproductive potential seen in female castes [34]—an idea that may apply to other social insects as well, and which may contribute to outcomes in the reproduction–immunity trade-off framework being difficult to predict.

Bumble bees are an excellent model system for studying trade-offs associated with ovary activation because the workers readily achieve ovary activation and egg laying [35], but the degree of activation varies among individuals within a colony (reviewed in Amsalem *et al*. [36]). Potential trade-offs associated with egg laying can therefore be decoupled from environmental and biological variables that necessarily covary with queen reproductive stage (i.e. season, age, mating status and diapause). In addition, the common eastern bumble bee (*Bombus impatiens*) is commercially available, and *B. impatiens* queens normally mate with a single drone (although not always) [37]. This means that workers within a colony are often highly genetically related (full sisters), offering a desirable experimental system for examining relationships between immunity and egg laying. Moreover, previous research has shown that in bumble bee workers, as shown in many solitary insects, immune activation does come with a fitness cost [38], which is one of the premises needed for a potential trade-off with reproduction to occur.

Previous research comparing immune gene expression in buff-tailed bumble bee (*Bombus terrestris*) queens at different reproductive stages suggests that a trade-off may be present: Colgan *et al*. show that during the non-reproductive diapause phase, expression of antimicrobial peptide immune effectors is high, and becomes lower as the queens transition out of diapause [39]. However, expression of other immune effectors first decreases during diapause, then increase again shortly after diapause [39]. Patterns of immune effector expression during the reproductive phase were not analysed, though, and have not been investigated in *B. impatiens.* Moreover, since the queen's reproductive stage and investment covaries with other factors (diapause, social environment, abiotic conditions and age, for example) additional approaches are necessary to investigate a reproductive–immunity trade-off from different angles.

Here, we investigate whether there is evidence for a reproductive–immunity trade-off in *B. impatiens*, another common bumble bee species. We do this through two complimentary methods: (i) comparing immune protein expression in queens at different reproductive stages, similar to Colgan *et al.* [39], and (ii) determining if the same patterns of immune protein expression linked to queen reproductive status are mirrored in reproductive and non-reproductive workers, a system that offers better control over extraneous variables.

## 2. Results

### (a) Queens

**Figure 1.**
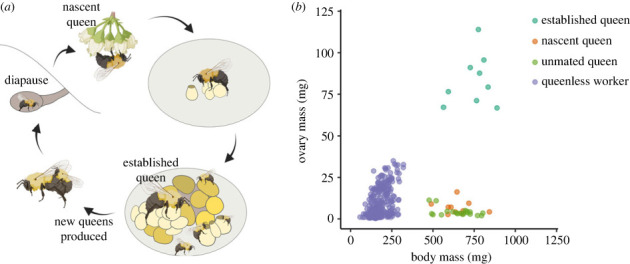
Overview of the bumble bee life cycle and anatomical patterns. (*a*) Bumble bee queens spend the winter in underground chambers. Upon emerging in the spring, nascent queens forage and search for a nesting site, but are not yet laying eggs. Once a nest is established, the colony grows through the spring into a few hundred individuals. In the summer, new queens and males are produced, mating occurs, and newly mated queens search for new overwintering sites. This figure was created using BioRender.com. (*b*) Relationships between ovary mass and body mass of female bumble bees.

We first investigated groups of queens at different life stages: unmated queens (approx. two weeks old, pre-diapause), nascent queens (approx. seven to eight months old, post-diapause) and established queens (approx. 10 months old, heading nests with greater than 100 workers) (illustrated in figure 1*a*). Despite not being able to distinguish potential differences due to age and environmental variables, these data provide important results to which our subsequent worker data can be compared. Ovary masses were significantly different between groups (Kruskal–Wallis test, *χ*^2^ = 23, *p* = 0.000010), which was clearly driven by the large ovaries of established queens (figure 1*b*). Ovary masses of unmated and nascent queens were similar (Welch's *t* test, *t* = −1.9, *p* = 0.083), which is consistent with the assumption that the nascent queens have not yet initiated egg laying. In contrast to ovary mass differences, body masses were comparatively stable, with only small magnitude and marginally significant differences identified (Kruskal–Wallis test to enable comparisons with ovary mass results: *χ*^2^ = 6.2, *p* = 0.046; or one-way ANOVA, as the data do meet the assumptions: *F* = 3.4, *p* = 0.043). When we subtracted the ovary mass from the body mass, we found no differences in non-ovary mass between groups (one-way ANOVA; *F* = 0.16, *p* = 0.85), indicating that non-ovary mass is relatively stable.

**Figure 2.**
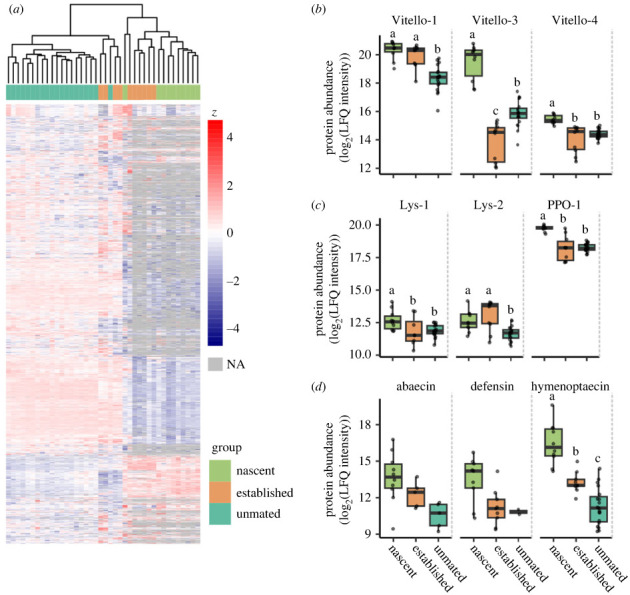
Haemolymph proteins in nascent, established and unmated queens. (*a*) Heat map of all identified protein groups identified in greater than 50% of samples (2284 in total). In total, 1000, 993 and 1419 were differentially expressed at 5% FDR (Benjamini–Hochberg correction) in established versus nascent, established versus unmated and nascent versus unmated groups, respectively. (*b*,*c*) Key proteins of interest. Different letters indicate statistical significance at 5% FDR. Abaecin and defensin were not evaluated statistically as they did not meet the threshold of being identified in greater than 50% of samples from all groups. Vitello-1, Vitello-3 and Vitello-4 refer to the vitellogenin isoforms A0A6P3DVD4, A0A6P3E5K4 and A0A6P3V1F4, respectively. Lys-1 and Lys-2 correspond to lysozyme isoforms A0A6P3DSG7 and A0A6P3DTW1, respectively. PPO stands for (pro)phenoloxidase-1.

Quantitative proteomics analysis of queen haemolymph shows that, of the 2923 identified protein groups (1% FDR) and 2284 quantified protein groups present in at least 20 out of 40 samples (figure 2*a*), 1000, 993 and 1419 were differentially expressed at 5% FDR (Benjamini–Hochberg correction) in established versus nascent, established versus unmated and nascent versus unmated groups, respectively. Hierarchical clustering of the samples showed that, with the exception of one sample, unmated queens formed a distinct cluster, while nascent queen samples tended to group with established queens. A large number of proteins were not identified in a fraction of the established and nascent queen samples (indicated as grey tiles figure 2*a*), indicating that they were either not present or were present below the limit of detection. This is a real biological phenomenon, and not a consequence of deteriorating instrument sensitivity, as the sample orders were randomized ahead of injection to avoid correlation with experimental groups.

While additional insights into queen life stage transitions may be gleaned from the multitude of differentially expressed and differentially identified proteins, we were specifically interested in vitellogenins and conserved immune proteins, owing to their central roles in reproduction and immunity. We therefore extracted data for all vitellogenins quantified in queens (vitellogenin-1, vitellogenin-3 and vitellogenin-4, which correspond to accession numbers A0A6P3DVD4, A0A6P3E5K4 and A0A6P3V1F4, respectively), as well as lysozyme-1 (A0A6P3DSG7), lysozyme-2 (A0A6P3DTW1), phenoloxidase 1 (A0A6P3UWM1), and the antimicrobial peptides abaecin (A0A6P3DU31), defensin-1 (A0A6P3DLY7) and hymenoptaecin (A0A6P3DYI5).

All the vitellogenins were among those significantly differentially expressed in queens, and all displayed the highest abundance in the nascent queens (figure 2*b*). Vitellogenin-1 had the lowest abundance in unmated queens, vitellogenin-3 had the lowest abundance in established queens, and vitellogenin-4 had similarly low amounts in unmated and established queens. Lysozyme-1, lysozyme-2 and phenoloxidase 1—immune effector enzymes—were also all among those differentially expressed, with high levels in nascent queens and low levels in unmated queens (figure 2*c*).

Among the antimicrobial peptides, hymenoptaecin was the only one passing the initial protein filtering cut-off of being identified in at least 50% of samples. It was strongly differentially expressed, again with the highest levels in the nascent queens, and the lowest levels in unmated queens (figure 2*d*). Abaecin and defensin-1 showed the same patterns of expression.

### (b) Workers

**Figure 3.**
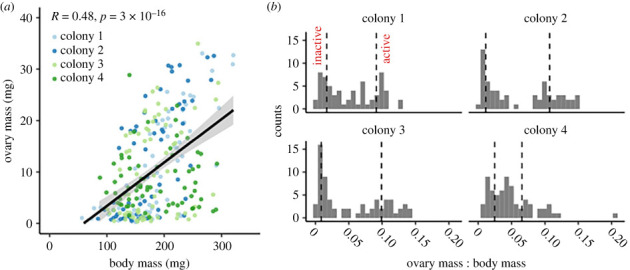
Sampling approach for reproductively active and inactive workers. (*a*) Ovary mass is a key parameter linked to active oogenesis, but it correlates significantly with body size. (*b*) The ovary mass-to-body mass ratio better indicates degree of reproductive activation. Dotted lines indicate the 1st and 4th quartile boundaries. Haemolymph samples from the corresponding reproductively active and inactive bees were subsequently analysed by proteomics.

Here, we used ovary mass relative to body size as a metric for reproductive activation, as ovary mass is a highly plastic trait associated with egg laying, whereas body mass is relatively stable (as we demonstrated in queens at different reproductive stages; figure 1*b*). This approach overcomes a problematic relationship we observed within the data: there was a highly significant correlation between ovary mass and body mass among queenless workers from all four colonies (Pearson correlation, *R* = 0.48, *p* = 3 × 10^−16^; figure 3*a*). Therefore, to categorize workers into those with ‘active' and ‘inactive' ovaries, we selected those with the highest and lowest (quartiles) ovary mass-to-body mass ratios (figure 3*b*), which allowed us to avoid picking those that had heavy ovaries simply as a consequence of a larger body mass. These quartile cut-offs were in the range of what was observed as biologically relevant for queens: that the highest ratio among nascent queens was 0.025 and the lowest ratio among established queens was 0.075.

We also inspected the ovary mass-to-body mass ratios of the 10 queenright workers to 10 randomly sampled workers which had been queenless for 10 days to confirm that this was a sufficient duration for ovary activation. The queenless worker ratios were higher and more variable than the queenright worker ratios (Wilcoxon rank-sum test, *W* = 4, *p* = 0.00058) indicating that ovary activation had commenced (electronic supplementary material, figure S1). This was corroborated by the observation of multiple egg clutches in the nest 10 days after de-queening.

**Figure 4.**
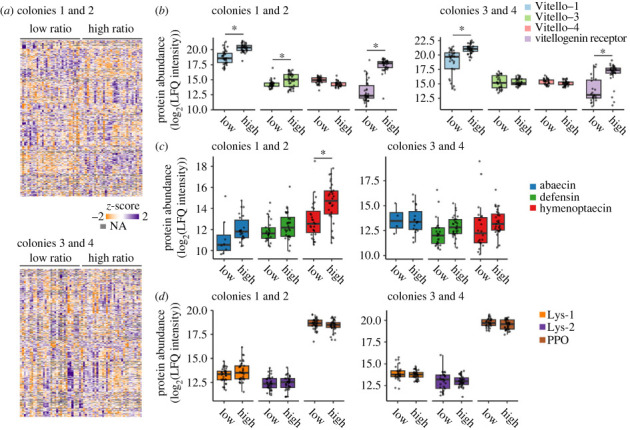
Proteomics results of reproductively active and inactive workers. (*a*) Phenotypic and proteomics data from colonies 1 and 2 were collected independently of colonies 3 and 4. (*a*) After filtering, 3259 and 3252 protein groups were quantified. Low ratio refers to samples from bees with low ovary mass-to-body mass ratios (reproductively inactive) and high ratio corresponds to high ovary mass-to-body mass ratios (reproductively active). In total, 772 and 532 proteins were significantly different between high ratio and low ratio groups at 5% FDR (Benjamini–Hochberg correction). (*b–d*) Expression patterns of key proteins. Asterisks indicate statistical significance at 5% FDR. Vitello-1, Vitello-3 and Vitello-4 refer to the vitellogenin isoforms A0A6P3DVD4, A0A6P3E5K4 and A0A6P3V1F4, respectively. Lys-1 and Lys-2 correspond to lysozyme isoforms A0A6P3DSG7 and A0A6P3DTW1, respectively. PPO stands for (pro)phenoloxidase-1.

We next compared patterns of protein expression in haemolymph from workers sorted into the high and low ovary mass-to-body mass ratio groups as described above. Of the 3605 and 3861 protein groups identified, 3259 and 3252 were quantified in batch 1 (colonies 1 and 2) and batch 2 (colonies 3 and 4), respectively (figure 4*a*). Differential expression analysis showed that, while accounting for source colony as a blocking factor, and sample injection order as a fixed factor covariate, 772 and 532 protein groups were differentially expressed between high and low ratio groups at 5% FDR (Benjamini–Hochberg correction). Our initial models included nest location (inside the canopy versus outside) as a covariate, since we observed that ovary masses of bees sampled from inside the canopy tended to be higher than those outside (electronic supplementary material, figure S2); however, this factor was not influential for protein expression data in worker batch 1 and was dropped from the model. It was influential for worker batch 2 data, with 149 significant proteins; therefore, in this model it was retained.

As conducted for queens previously, we examined expression patterns of vitellogenins and immune proteins specifically, in addition to vitellogenin receptor (which was not quantified in the queen dataset). We found that in both sample batches, vitellogenin-1 and the vitellogenin receptor were differentially expressed, with higher levels in the high ratio groups (figure 4*b*). Vitellogenin-3 also displayed higher levels in the high ratio group of batch 1, but not batch 2.

Next, we inspected expression patterns of immune proteins. Under the assumption of a reproduction–immunity trade-off, one would expect the samples belonging to the high ratio group to have lower constitutive expression of immune proteins. However, we observed no differences in lysozyme-1, lysozyme-2, or phenoloxidase 1 between high and low ratio groups for either batch. Among the antimicrobial peptides, the only significant difference observed was for hymenoptaecin in batch 1, and this showed the opposite pattern to what would be expected under the trade-off hypothesis: higher levels in the high ratio group. This protein was not significantly different among groups in batch 2. Consistent with these observations, this protein was also expressed at higher levels in queenless workers (which have smaller ovaries) compared to queenright workers (which have larger ovaries) (electronic supplementary material, figure S3 and data S2).

## 3. Discussion

While the patterns of differential expression observed in *B. impatiens* queens are largely consistent with what would be predicted under a reproduction–immunity resource allocation trade-off (higher levels of constitutive immune proteins in nascent, pre-reproductive queens), the data from workers across a gradient of ovary activation are not. We argue that comparing proteins expressed in high and low ovary mass-to-body mass ratio groups of workers offers more control for assessing such a trade-off, since these samples have reduced environmental and genetic differences. As seen in other systems [4–8], the act of mating itself may also induce physiological changes that complicate data interpretation. Conversely, the queen sample groups, while ostensibly supporting a trade-off, unavoidably confound with season, age and social environment.

The tendency for nascent queens to express the highest constitutive levels of immune proteins, though consistent with a trade-off, may instead be a consequence of differences in selective pressure during non-reproductive periods of their life cycle and not due to their reproductive status. For example, nascent queens have recently undergone a period of diapause, during which they are immobile and may experience large ambient temperature fluctuations, and some research points to cross-talk between cold stress and immunity in insects [40]. Furthermore, unlike established queens, nascent queens are solely responsible for foraging, at which time they may encounter new pathogens (e.g. on flowers [41]). One may argue that diapausing and nascent queens are at lower risk of pathogen contact than established queens due to their temporary solitude; however, they are under more intense selective pressure to withstand challenges, since succumbing to an infection, for example, would be the end of her genetic lineage. Conversely, if an established queen perishes, the colony that remains will first rear new queens from remaining fertilized eggs, and then drones from worker reproduction, so the queen's genes can thus still persist.

We focused on vitellogenins and immune effectors in our proteomics analysis to examine whether their expression patterns supported the existence of a trade-off. Vitellogenin is an egg yolk protein precursor, but there are four distinct vitellogenin and vitellogenin-like genes in bumble bees [42] and the proteins are likely multifunctional, as has been shown in other social insects [43]. Differences in the proteins' domains indicate that they likely have distinct functions, but they have not yet been clearly defined. We observed three of the four vitellogenins in our dataset, and the high levels of vitellogenin-3 and vitellogenin-4 we observed in nascent queen haemolymph and subsequent decrease in established queen haemolymph is consistent with uptake by developing oocytes, representing the expected diversion of resources as queens transition from having inactive to active ovaries.

Under a resource allocation trade-off, and assuming that a decrease in circulating vitellogenin is indicative of increased uptake by oocytes, one would expect constitutive immune protein expression to decrease as circulating vitellogenins decrease. However, while the concurrent low levels of lysozyme-1, phenoloxidase 1, and antimicrobial peptides in established queens is consistent with a trade-off with reproduction, lysozyme-2 levels are unchanged between nascent and established queens, which is at odds with the predicted pattern. Moreover, the consistently low levels of immune enzymes and antimicrobial peptides in unmated, non-reproductive queens are likely not linked to their reproductive status, but to their young age, as their immune repertoire has likely not had sufficient time to develop (as also suggested previously in *B. terrestris* [39]), highlighting again the problem of confounding variables.

The high abundance of circulating vitellogenins in nascent (post-diapause) queen haemolymph aligns with what has previously been observed in *B. terrestris* queens. Colgan *et al*. [39] reported that circulating vitellogenin rapidly increases in abundance after diapause (as much as fivefold in as little as 48 h post-diapause) in *B. terrestris*. Colgan *et al*. [39] also observed that unmated queens had the lowest levels of abaecin, defensin-1 and hymenoptaecin, which then increased after mating and remained high during diapause, to finally decrease again 48 h after diapause. The authors suggest that high levels of antimicrobial peptides may help increase likelihood of queen survival while other biological functions, including reproduction, are shut down. Though this could be interpreted as evidence for a trade-off, the lack of association between immune proteins and worker reproduction in our study suggests that the expression patterns in queens may actually be dictated by the fitness benefit gained from improved immunity during this vulnerable stage.

Unlike the queen samples, confounding effects on the worker samples were minimized (although in the future, worker age should be controlled more precisely). The observation that in both batches of worker samples, the high ratio group yielded higher levels of vitellogenin-1 and vitellogenin receptor than the low ratio groups indicates that our high and low ratio groups were dividing the population in a biologically relevant way. We were surprised to see that the constitutive levels of immune proteins were either unchanged between ratio groups or showed opposite patterns to what would be expected under a trade-off (higher levels in the reproductively active group). The apparent lack of trade-off suggests that the observed patterns in queens are due to extraneous variables or alternate selective pressures.

Future experiments should go beyond analysing constitutive expression of immune proteins, as we have conducted here, and additional factors, such as worker age or aggressive behaviour, should be more tightly accounted for. In our experiments, we opted to minimize disturbance due to sampling events for each colony, at a cost of not obtaining data on the specific age of each worker in our analysis. While we did avoid sampling callow (very young) bees and accounted for the influence of within-nest location at the time of sampling (inside versus outside the canopy), the influence of age on ovary activation and protein expression should be investigated further in the future. Testing the ability for an individual to launch a concerted immune response after stimulation with a pathogen or other elicitor (i.e. immune adjustability) will also provide a more complete picture of actual fitness outcomes, and may help establish relationships between constitutive expression and effectual tolerance or resistance.

### (a) Conclusion

These results collectively suggest that results of investigations into reproduction–immunity trade-offs should be interpreted cautiously when they rely primarily on comparisons between life stages or pre-to-post mating transitions. Especially for hymenopterans, but also other species, life stages and mating status may confound with variables not explicitly linked to reproduction, and the two cannot always be distinguished.

## 4. Methods

### (a) Queens

Queens were sourced from commercially produced *B. impatiens* colonies (BioBest) as well as field sites where high-densities of queens overwinter (see electronic supplementary material, data S1 for complete sample descriptions). In total, 10 established queens (nine from BioBest, heading nests with approx. 200 workers, and one that was caught in the field and allowed to establish a nest of greater than 100 workers), 10 nascent queens (just emerged from overwintering sites), and 20 unmated queens (produced from de-queened BioBest colonies) were analysed. Of the queens sourced from BioBest, two were from colonies purchased in March and seven were from colonies purchased in May. Half of the established queens were sampled by briefly exposing the entire colony to carbon dioxide gas and retrieving the queen, and the other half were sampled without using anaesthetic, but all were exposed to carbon dioxide before freezing. The nascent, overwintered queens were sampled from an approx. 3 m by 15 m area adjacent to a commercial greenhouse operation. The nascent queens were caught on 10 April 2022 and transported to the laboratory, where they were briefly anaesthetized with carbon dioxide and frozen at −70°C. Four of the de-queened BioBest colonies were allowed to rear new queens, which were sampled approximately 30 days after de-queening (June). This is a regular process within bumble bee colonies: if the foundress queen is removed from the colony or dies, this stimulates the workers to rear a subset of the existing diploid eggs or young larvae into new gynes ahead of the normal seasonal timing of gyne-rearing [44]. The new, unmated queens were obtained as they exited the nest (five per colony) by temporarily changing the nest entrance from a queen-excluding position (such that queens cannot exit the colony) to a queen-passing position (that allows the queens to exit). This allowed us to be sure the foundress queen was not accidentally sampled, and that the new queens were of a suitable age to begin searching for a mate (at approx. one week old [45]). The queens were then also anaesthetized and frozen until dissection. Apart from the unmated queens which were reared from the same colony, genetic relatedness between queens is not known.

Prior to dissection, queen mass was recorded on an analytical balance. Then, one hind leg was removed at the coxa and the bee was allowed to thaw, which produces a small drop of haemolymph exuding from the thorax without risk of contamination from other organs. This haemolymph was collected with a pipet and dispensed into a tube containing 50 µl of ammonium bicarbonate buffer (50 mM) kept on ice. The bee was then pinned and the ventral surface of the abdomen was removed to expose the ovaries, which were removed and placed in a pre-weighed tube. The tube containing the ovaries was then weighed again and the difference (ovary mass) was recorded.

### (b) Workers

Workers were sourced from four queenless colonies—two originating from BioBest and two originating from queens caught at the same field collection site where the nascent queens were obtained. We induced queenlessness prior to worker collection because in *B. impatiens,* unlike some other bumble bee species, workers rarely reproduce in the presence of a queen and only about 9–11% of workers have well-developed ovaries even under queenless conditions [46,47]. We used an experimental design where one researcher collected data and processed samples from one batch of colonies (the two of BioBest origin), followed by another researcher following the same methods on another batch of colonies from a different origin (the two colonies originating from field collections). This allowed us to evaluate consistency of results from independent datasets. The four colonies from which workers were sourced were not among those used for producing unmated queens described above, but, with the exception of one colony for which the queen was not retained, the queens removed from these colonies were included within the ‘established' queen group described above.

The two BioBest colonies used for worker collections were purchased on 1 March 2022, and dequeened shortly after acquisition while the colonies were still in the population growth phase and before they began rearing new reproductives. We did not count all workers in the colony, but we estimate that there were approximately 200 individuals. Other than the foundress queen, there were only workers present. Five workers were first removed from each colony, comprising the 10 queenright worker samples. Ten days after removing the queen, the whole nest was anaesthetized and workers were sampled, with those located under the nest canopy separated from those outside the nest canopy. We took this approach because some data suggest that reproductive workers may be spatially organized [47,48], and we wished to account for nest location in our proteomics analysis. Previous research has recorded that, in queenless *B. impatiens* colonies, worker ovaries become fully developed within 7 or 8 days [46,47], corresponding to shortly before our sampling date. Callow workers with silvery grey appearance were avoided, since very young bees are not capable of laying eggs and thus are not biologically relevant for our study system. Abundant egg clutches were observed, confirming that this time frame was sufficient for worker ovary activation.

The two colonies originating from field-collected queens were produced by providing the queens with a nesting box, constant food (pollen replaced every other day, and 50% sucrose solution fed through a wick feeder). Two young workers obtained from the BioBest colonies were added to the nesting box to help stimulate the queen to begin laying eggs. On 17 June, once the colonies had expanded their populations considerably (i.e. they were in the population growth phase and not yet rearing drones or queens naturally, similar to the BioBest colonies described above), the colonies were de-queened. We estimate that the total population was slightly smaller than the BioBest colonies, but still with greater than 100 individuals per colony, and, similar to the BioBest colonies, no reproductives other than the founding queen were present. Ten days later, workers were sampled as previously described for the two BioBest colonies. All workers were anaesthetized, frozen, then weighed and dissected as described for queens. A total of 63, 64, 67 and 67 workers were weighed and dissected for colonies 1, 2, 3 and 4, respectively.

### (c) Worker haemolymph sample selection

To sort workers into groups with activated and inactivated ovaries, we took the ratio of ovary mass-to-body mass and selected samples from the lower and upper quartiles as our two experimental groups. This ensured that we did not select samples from bees that had large ovaries simply as a consequence of their body size. High and low ratio samples from colonies 1 and 2 (68 total) were processed in a single batch, and samples from colonies 3 and 4 (64 total) were processed in a separate batch.

### (d) Sample preparation for proteomics

Haemolymph proteins were precipitated by adding ice-cold acetone to a final concentration of 80%. Samples were then incubated overnight at −20°C, and the precipitate was pelleted by centrifugation at 10 000*g* for 15 min (4°C). The supernatant was discarded, and the pellet was washed twice with cold, 80% acetone, discarding the wash. The pellet was then allowed to air dry for 15 min, at which time it was resuspended in 25 µl of digestion buffer (8 M urea, 2 M thiourea, 100 mM Tris, pH 8.0). Protein concentration was determined using a Bradford assay, or, in cases where sample amount was very limited, the amount was assumed to be 10 µg. Approximately 10 µg of protein was then reduced (0.2 µg of dithiothreitol, 30 min), alkylated (1 µg of iodoacetamide, dark, 30 min) and digested (0.4 µg of Lys-C/Trypsin mix, Promega). After 4 h of digestion in the urea buffer, 250 µl of 50 mM ammonium bicarbonate buffer was added and samples were allowed to digest overnight at room temperature. The samples were acidified to pH less than 2.0 with 20% formic acid and desalted using in-house made C18 STAGE tips [49]. After loading the sample, the STAGE tips were washed with 3 × 250 µl buffer A (0.5% acetonitrile, 0.5% formic acid, in water), then eluted with 200 µl elution buffer (40% acetonitrile, 0.5% formic acid). Samples were evaporated to dryness using a speed-vac (approx. 2 h, room temperature) and suspended in 11 µl buffer A. Every sample was checked using a nanodrop to determine peptide concentration and verify the absence of absorbance at 240 nm (which would indicate residual digestion buffer contamination). Samples were diluted to a final concentration of 18.75 ng µl^–1^.

### (e) Liquid chromatography and mass spectrometry

A total of 75 ng of digested peptides were injected onto the LC system in randomized order. The digest was separated using NanoElute UHPLC system (Bruker Daltonics) with Aurora Series Gen2 (CSI) analytical column (25 cm × 75 µm 1.6 µm FSC C18, with Gen2 nanoZero and CSI fitting; Ion Opticks, Parkville, Victoria, Australia) heated to 50°C and coupled to timsTOF Pro (Bruker Daltonics) operated in DIA-PASEF mode. A standard 30 min gradient was run from 2% B to 12% B over 15 min, then to 33% B from 15 to 30 min, then to 95% B over 0.5 min, and held at 95% B for 7.72 min, where buffer B consisted of 0.1% formic acid in 99.4% acetonitrile and buffer A consisted of 0.1% aqueous formic acid and 0.5% acetonitrile in water. Before each run, the analytical column was conditioned with four column volumes of buffer A. The NanoElute thermostat temperature was set at 7°C. The analysis was performed at 0.3 µl min^−1^ flow rate.

The trapped ion mobility-time of flight mass spectrometer (TimsTOF Pro; Bruker Daltonics, Germany) was set to parallel accumulation-serial fragmentation (PASEF) scan mode for data-independent acquisition scanning (100–1700 *m/z*). The capillary voltage was set to 1800 V, drying gas to 3 l/min and drying temperature to 180°C. The MS1 scan was followed by 17 consecutive PASEF ramps containing 22 non-overlapping 35 *m/z* isolation windows (table 1), covering the 319.5–1089.5 *m/z* range. As for TIMS setting, ion mobility range (1/k_0_) was set to 0.70–1.35 V·s cm^−2^, 100 ms ramp time and accumulation time (100% duty cycle), and ramp rate of 9.42 Hz; this resulted in 1.91 s of total cycle time. The collision energy was ramped linearly as a function of mobility from 27 eV at 1/*k*_0_ = 0.7 V·s cm^−2^ to 55 eV at 1/*k*_0_ = 1.35 V·s cm^−2^. Mass accuracy was typically within 3 ppm and is not allowed to exceed 7 ppm. For calibration of ion mobility dimension, the ions of Agilent ESI-Low Tuning Mix ions were selected (*m/z* [Th], 1/*k*_0_ [Th]: 622.0290, 0.9915; 922.0098, 1.1986; 1221.9906, 1.3934). The TimsTOF Pro was run with TimsControl 3.0.0 (Bruker), and the LC and MS were controlled with HyStar 6.0 (Bruker).

**Table 1.** DIA PASEF isolation windows.

#MS type	cycle Id	start IM [1/K_0_]	end IM [1/K_0_]	start mass [*m/z*]	end mass [*m/z*]
MS1	0	–	–	–	–
PASEF	1	0.9614	1.2687	914.5	949.5
PASEF	1	0.7	0.9427	319.5	354.5
PASEF	2	0.9806	1.2879	949.5	984.5
PASEF	2	0.7	0.9618	354.5	389.5
PASEF	3	0.9998	1.3071	984.5	1019.5
PASEF	3	0.7	0.981	389.5	424.5
PASEF	4	1.0189	1.3262	1019.5	1054.5
PASEF	4	0.7	1.0002	424.5	459.5
PASEF	5	1.0381	1.3454	1054.5	1089.5
PASEF	5	0.7121	1.0194	459.5	494.5
PASEF	6	0.7312	1.0385	494.5	529.5
PASEF	7	0.7504	1.0577	529.5	564.5
PASEF	8	0.7696	1.0769	564.5	599.5
PASEF	9	0.7888	1.0961	599.5	634.5
PASEF	10	0.808	1.1153	634.5	669.5
PASEF	11	0.8271	1.1344	669.5	704.5
PASEF	12	0.8463	1.1536	704.5	739.5
PASEF	13	0.8655	1.1728	739.5	774.5
PASEF	14	0.8847	1.192	774.5	809.5
PASEF	15	0.9039	1.2112	809.5	844.5
PASEF	16	0.923	1.2303	844.5	879.5
PASEF	17	0.9422	1.2495	879.5	914.5

### (f) Data processing

The data were searched using DIA-NN version 1.8.1 [50] with the default parameters except that the options ‘FASTA digest for library-free search', ‘Deep learning-based spectra', 'RTs and IMs prediction', ‘unrelated runs' and ‘MBR’ were checked, ‘Protein inference' was set to protein names from FASTA, two missed cleavages were allowed, and ‘Neural network classifier' was set to double-pass mode. The FASTA database was downloaded from Uniprot on 5 December 2022 (19 175 entries) and a comprehensive list of potential protein contaminants was appended to the database [51]. Data for queens (40 samples), worker batch 1 (68 samples) and worker batch 2 (64 samples) were searched independently as the raw data became available. In total, 2923, 3605 and 3861 unique protein groups were identified in each search, after removing reverse hits and contaminants, at 1% protein and peptide false discovery rates.

### (g) Statistical analysis

All data processing and statistical tests were performed in R (v. 4.3.0) using R Studio (v. 2023.03.1 + 446) [52]. Queen ovary mass data were non-normal and variance between groups was unequal; therefore, a Kruskal–Wallis test was used for this parameter. Queen body mass data were normal with equal variance; therefore, an ANOVA was used alongside a Kruskal–Wallis test in order to draw comparisons between statistical results of ovary mass and body mass analyses. A linear model was used to evaluate worker ovary mass as a function of body mass (continuous) and colony (categorical, four levels), and residuals were inspected for appropriateness of fit using tools within the DHARMa package [53]. All plots were produced using ggplot2 [54]. In a preliminary analysis, we observed a trend for workers sampled from inside the nest canopy to have higher ovary mass-to-body mass ratios than those sampled from outside the nest (electronic supplementary material, figure S2); therefore, we accounted for this variable in the initial analysis of the proteomics data (see below).

Proteomics data were analysed using limma [55]. The data were first log2 transformed, then protein groups were filtered to remove those identified in fewer than 50% of samples for the queen dataset, and fewer than 25% of samples for the worker datasets (since the worker datasets have a much larger sample size than the queen dataset, relaxing the filtering stringency to 25% still yields sample sizes large enough to draw meaningful comparisons). This left a total of 2284, 3259 and 3252 protein groups considered to be quantified in the queen, worker batch 1 and worker batch 2 datasets, respectively. For worker samples, differential expression analysis was then performed using ratio group (categorical, two levels: high and low), nest location (categorical, two levels: inside nest canopy and outside nest canopy) and sample injection order (continuous) as fixed factors, and colony as a blocking variable. For batch 1, nest location was determined to be non-influential, so this term was removed from the final model. For batch 2, nest location was influential, and retained in the final model. Protein groups were considered to be differentially expressed if their adjusted *p*-value (based on the Benjamini–Hochberg correction) was below 0.05. For queen samples, the differential expression model included queen stage (categorical, three levels: established, nascent and unmated) and injection order (continuous) as fixed factors. We have not conducted a GO term enrichment analysis in the present work because of our *a priori* interest in a small number of specific proteins, some of which (hymenoptaecin and abaecin isoforms) are not associated with any GO terms for molecular processes.

## Data Availability

Ovary mass data for workers and queens are supplied in electronic supplementary material, data S1. All raw proteomics data, proteomics sample metadata, proteomics search results, and FASTA databases are available on the MassIVE proteomics data archive (MSV000091414, accessible at www.massive.ucsd.edu). The protein data upon which differential expression was performed, metadata, and statistical outcomes are reported in electronic supplementary material, data S1 (data referred to in the main text) and electronic supplementary material, data S2 (data referred to in electronic supplementary material, figure S30) for easy access. The R codes underlying all statistical analyses are available in electronic supplementary material File S1, which contains the script for limma analysis, phenotype analysis and immune protein analysis. Supplementary material is available online [56].

## References

[1] Williams GC. 1966 Natural selection, the costs of reproduction, and a refinement of Lack's principle. Am. Nat. **100**, 687-690. (10.1086/282461)

[2] Schwenke RA, Lazzaro BP, Wolfner MF. 2016 Reproduction–immunity trade-offs in insects. Annu. Rev. Entomol. **61**, 239-256. (10.1146/annurev-ento-010715-023924) 26667271 PMC5231921

[3] McNamara KB, Van Lieshout E, Jones TM, Simmons LW. 2013 Age-dependent trade-offs between immunity and male, but not female, reproduction. J. Anim. Ecol. **82**, 235-244. (10.1111/j.1365-2656.2012.02018.x) 22849327

[4] Baer B, Armitage SA, Boomsma JJ. 2006 Sperm storage induces an immunity cost in ants. Nature **441**, 872-875. (10.1038/nature04698) 16778889

[5] Bascuñán-García AP, Lara C, Córdoba-Aguilar A. 2010 Immune investment impairs growth, female reproduction and survival in the house cricket, *Acheta domesticus*. J. Insect. Physiol. **56**, 204-211. (10.1016/j.jinsphys.2009.10.005) 19840805

[6] Fedorka KM, Linder JE, Winterhalter W, Promislow D. 2007 Post-mating disparity between potential and realized immune response in *Drosophila melanogaster*. Proc. R. Soc. B **274**, 1211-1217. (10.1098/rspb.2006.0394) PMC2189566 17311779

[7] Short SM, Wolfner MF, Lazzaro BP. 2012 Female *Drosophila melanogaster* suffer reduced defense against infection due to seminal fluid components. J. Insect. Physiol. **58**, 1192-1201. (10.1016/j.jinsphys.2012.06.002) 22698822 PMC3423548

[8] Siva-Jothy MT, Tsubaki Y, Hooper RE. 1998 Decreased immune response as a proximate cost of copulation and oviposition in a damselfly. Physiol. Entomol. **23**, 274-277. (10.1046/j.1365-3032.1998.233090.x)

[9] Jehan C, Sabarly C, Rigaud T, Moret Y. 2022 Senescence of the immune defences and reproductive trade-offs in females of the mealworm beetle, *Tenebrio molitor*. Sci. Rep. **12**, 19747. (10.1038/s41598-022-24334-y) 36396809 PMC9671880

[10] Ahmed AM, Hurd H. 2006 Immune stimulation and malaria infection impose reproductive costs in *Anopheles gambiae* via follicular apoptosis. Microbes Infect. **8**, 308-315. (10.1016/j.micinf.2005.06.026) 16213176

[11] Ahmed A, Baggott S, Maingon R, Hurd H. 2002 The costs of mounting an immune response are reflected in the reproductive fitness of the mosquito *Anopheles gambiae*. Oikos **97**, 371-377. (10.1034/j.1600-0706.2002.970307.x)

[12] Kelly CD. 2011 Reproductive and physiological costs of repeated immune challenges in female Wellington tree weta (Orthoptera: Anostostomatidae). Biol. J. Linn. Soc. **104**, 38-46. (10.1111/j.1095-8312.2011.01714.x)

[13] McKean KA, Yourth CP, Lazzaro BP, Clark AG. 2008 The evolutionary costs of immunological maintenance and deployment. BMC Evol. Biol. **8**, 1-19. (10.1186/1471-2148-8-76) 18315877 PMC2292698

[14] Nystrand M, Dowling DK. 2014 Dose-dependent effects of an immune challenge at both ultimate and proximate levels in *Drosophila melanogaster*. J. Evol. Biol. **27**, 876-888. (10.1111/jeb.12364) 24731072

[15] Reaney LT, Knell RJ. 2010 Immune activation but not male quality affects female current reproductive investment in a dung beetle. Behav. Ecol. **21**, 1367-1372. (10.1093/beheco/arq139)

[16] Stahlschmidt ZR, Rollinson N, Acker M, Adamo SA. 2013 Are all eggs created equal? Food availability and the fitness trade-off between reproduction and immunity. Funct. Ecol. **27**, 800-806. (10.1111/1365-2435.12071)

[17] Chérasse S, Aron S. 2018 Impact of immune activation on stored sperm viability in ant queens. Proc. Biol. Sci. **285**, 20182248. 30963911 10.1098/rspb.2018.2248 PMC6304054

[18] Kennedy A, Herman J, Rueppell O. 2021 Reproductive activation in honeybee (*Apis mellifera*) workers protects against abiotic and biotic stress. Phil. Trans. R. Soc. B **376**, 20190737. (10.1098/rstb.2019.0737) 33678021 PMC7938169

[19] Chan QW, Chan MY, Logan M, Fang Y, Higo H, Foster LJ 2013 Honey bee protein atlas at organ-level resolution. Genome Res. **23**, 1951-1960. (10.1101/gr.155994.113) 23878156 PMC3814894

[20] Gálvez D, Chapuisat M. 2014 Immune priming and pathogen resistance in ant queens. Ecol. Evol. **4**, 1761-1767. (10.1002/ece3.1070) 24963375 PMC4063474

[21] Domanitskaya EV, Liu H, Chen S, Kubli E. 2007 The hydroxyproline motif of male sex peptide elicits the innate immune response in *Drosophila* females. FEBS J. **274**, 5659-5668. (10.1111/j.1742-4658.2007.06088.x) 17922838

[22] Lawniczak MK, Begun DJ. 2004 A genome-wide analysis of courting and mating responses in *Drosophila melanogaster* females. Genome **47**, 900-910. (10.1139/g04-050) 15499404

[23] McGraw LA, Gibson G, Clark AG, Wolfner MF. 2004 Genes regulated by mating, sperm, or seminal proteins in mated female *Drosophila melanogaster*. Curr. Biol. **14**, 1509-1514. (10.1016/j.cub.2004.08.028) 15324670

[24] Peng J, Zipperlen P, Kubli E. 2005 *Drosophila* sex-peptide stimulates female innate immune system after mating via the Toll and Imd pathways. Curr. Biol. **15**, 1690-1694. (10.1016/j.cub.2005.08.048) 16169493

[25] Rueppell O, Aumer D, Moritz RF. 2016 Ties between ageing plasticity and reproductive physiology in honey bees (*Apis mellifera*) reveal a positive relation between fecundity and longevity as consequence of advanced social evolution. Curr. Opin. Insect Sci. **16**, 64-68. (10.1016/j.cois.2016.05.009) 27720052 PMC5094365

[26] Blacher P, Huggins TJ, Bourke AF. 2017 Evolution of ageing, costs of reproduction and the fecundity–longevity trade-off in eusocial insects. Proc. R. Soc. B **284**, 20170380. (10.1098/rspb.2017.0380) PMC5524490 28701554

[27] Rodrigues MA, Flatt T. 2016 Endocrine uncoupling of the trade-off between reproduction and somatic maintenance in eusocial insects. Curr. Opin. Insect Sci. **16**, 1-8. (10.1016/j.cois.2016.04.013) 27720042

[28] McAfee A, Chapman A, Pettis JS, Foster LJ, Tarpy DR. 2021 Trade-offs between sperm viability and immune protein expression in honey bee queens (*Apis mellifera*). Commun. Biol. **4**, 48. (10.1038/s42003-020-01586-w) 33420325 PMC7794525

[29] Castella G, Christe P, Chapuisat M. 2009 Mating triggers dynamic immune regulations in wood ant queens. J. Evol. Biol. **22**, 564-570. (10.1111/j.1420-9101.2008.01664.x) 19170815

[30] Shih S, Huntsman E, Flores M, Snow J. 2020 Reproductive potential does not cause loss of heat shock response performance in honey bees. Sci. Rep. **10**, 19610. (10.1038/s41598-020-74456-4) 33184302 PMC7661715

[31] Pamminger T, Treanor D, Hughes WO. 2016 Pleiotropic effects of juvenile hormone in ant queens and the escape from the reproduction–immunocompetence trade-off. Proc. R. Soc. B **283**, 20152409. (10.1098/rspb.2015.2409) PMC4721097 26763704

[32] Corona M, Velarde RA, Remolina S, Moran-Lauter A, Wang Y, Hughes KA, Robinson GE. 2007 Vitellogenin, juvenile hormone, insulin signaling, and queen honey bee longevity. Proc. Natl Acad. Sci. USA **104**, 7128-7133. (10.1073/pnas.0701909104) 17438290 PMC1852330

[33] Luo W et al. 2021 Juvenile hormone signaling promotes ovulation and maintains egg shape by inducing expression of extracellular matrix genes. Proc. Natl Acad. Sci. USA **118**, e2104461118. (10.1073/pnas.2104461118) 34544864 PMC8488625

[34] Hartfelder K, Guidugli-Lazzarini KR, Cervoni MS, Santos DE, Humann FC. 2015 Old threads make new tapestry—rewiring of signalling pathways underlies caste phenotypic plasticity in the honey bee, *Apis mellifera* L. In Advances in insect physiology, vol. 48 (eds A Zayed, CF Kent), pp. 1-36. London, UK: Academic Press.

[35] Bourke AF. 1988 Worker reproduction in the higher eusocial Hymenoptera. Q. Rev. Biol. **63**, 291-311. (10.1086/415930)

[36] Amsalem E, Grozinger CM, Padilla M, Hefetz A. 2015 The physiological and genomic bases of bumble bee social behaviour. In Advances in insect physiology, vol. 48 (eds A Zayed, CL Kent), pp. 37-93. London, UK: Academic Press.

[37] Payne C, Laverty T, Lachance M. 2003 The frequency of multiple paternity in bumble bee (*Bombus*) colonies based on microsatellite DNA at the B10 locus. Insectes Soc. **50**, 375-378. (10.1007/s00040-003-0692-2)

[38] Moret Y, Schmid-Hempel P. 2000 Survival for immunity: the price of immune system activation for bumblebee workers. Science **290**, 1166-1168. (10.1126/science.290.5494.1166) 11073456

[39] Colgan TJ, Finlay S, Brown MJ, Carolan JC. 2019 Mating precedes selective immune priming which is maintained throughout bumblebee queen diapause. BMC Genomics **20**, 1-18. (10.1186/s12864-019-6314-9) 31823732 PMC6902353

[40] Sinclair BJ, Ferguson LV, Salehipour-Shirazi G, MacMillan HA. 2013 Cross-tolerance and cross-talk in the cold: relating low temperatures to desiccation and immune stress in insects. Integr. Comp. Biol. **53**, 545-556. (10.1093/icb/ict004) 23520401

[41] Burnham PA, Burnham PA, Alger SA, Case B, Boncristiani H, Hébert-Dufresne L, Brody AK. 2021 Flowers as dirty doorknobs: deformed wing virus transmitted between *Apis mellifera* and *Bombus impatiens* through shared flowers. J. Appl. Ecol. **58**, 2065-2074. (10.1111/1365-2664.13962)

[42] Zhao F, Morandin C, Jiang K, Su T, He B, Lin G, Huang Z 2021 Molecular evolution of bumble bee vitellogenin and vitellogenin-like genes. Ecol. Evol. **11**, 8983-8992. (10.1002/ece3.7736) 34257940 PMC8258195

[43] Amdam GV, Norberg K, Hagen A, Omholt SW. 2003 Social exploitation of vitellogenin. Proc. Natl Acad. Sci. USA **100**, 1799-1802. (10.1073/pnas.0333979100) 12566563 PMC149913

[44] Hefetz A, Robinson GE, Huang Z-Y., Borst DW, Cnaani J. 1997 Caste determination in *Bombus terrestris*: differences in development and rates of JH biosynthesis between queen and worker larvae. J. Insect. Physiol. **43**, 373-381. (10.1016/S0022-1910(96)00106-0) 12769899

[45] Röseler P-F, Van Honk CG. 1990 Castes and reproduction in bumblebees. In *Social insects: an evolutionary approach to castes and reproduction*, pp. 147-166. Berlin, Germany: Springer.

[46] Cnaani J, Schmid-Hempel R, Schmidt J. 2002 Colony development, larval development and worker reproduction in *Bombus impatiens* Cresson. Insectes Soc. **49**, 164-170. (10.1007/s00040-002-8297-8)

[47] Jandt JM, Dornhaus A. 2011 Competition and cooperation: bumblebee spatial organization and division of labor may affect worker reproduction late in life. Behav. Ecol. Sociobiol. **65**, 2341-2349. (10.1007/s00265-011-1244-9)

[48] Jandt JM, Dornhaus A. 2009 Spatial organization and division of labour in the bumblebee *Bombus impatiens*. Anim. Behav. **77**, 641-651. (10.1016/j.anbehav.2008.11.019)

[49] Rappsilber J, Ishihama Y, Mann M. 2003 Stop and go extraction tips for matrix-assisted laser desorption/ionization, nanoelectrospray, and LC/MS sample pretreatment in proteomics. Anal. Chem. **75**, 663-670. (10.1021/ac026117i) 12585499

[50] Demichev V, Messner CB, Vernardis SI, Lilley KS, Ralser M. 2020 DIA-NN: neural networks and interference correction enable deep proteome coverage in high throughput. Nat. Methods **17**, 41-44. (10.1038/s41592-019-0638-x) 31768060 PMC6949130

[51] Frankenfield AM, Ni J, Ahmed M, Hao L. 2022 Protein contaminants matter: building universal protein contaminant libraries for DDA and DIA proteomics. J. Proteome Res. **21**, 2104-2113. (10.1021/acs.jproteome.2c00145) 35793413 PMC10040255

[52] R Core Team. 2021 R: a language and environment for statistical computing. Vienna, Austria: R Foundation for Statistical Computing.

[53] Hartig F. 2022 DHARMa: Residual Diagnostics for Hierarchical (Multi-Level/Mixed) Regression Models. R package version 0.4.6. See https://CRAN.R-project.org/package=DHARMa.

[54] Villanueva RAM, Chen ZJ. 2019 Ggplot2: elegant graphics for data analysis. New York, NY: Taylor & Francis.

[55] Ritchie ME, Phipson B, Wu D, Hu Y, Law CW, Shi W, Smyth GK. 2015 limma powers differential expression analyses for RNA-sequencing and microarray studies. Nucleic Acids Res. **43**, e47. (10.1093/nar/gkv007) 25605792 PMC4402510

[56] McAfee A, Chapman A, Bao G, Tarpy DR, Foster LJ. 2024 Investigating trade-offs between ovary activation and immune protein expression in bumble bee (*Bombus impatiens*) workers and queens. Figshare*.* (10.6084/m9.figshare.c.7021299)

